# Design and rationale of a large, international, prospective cohort study to evaluate the occurrence of malformations and perinatal/neonatal death using insulin detemir in pregnant women with diabetes in comparison with other long-acting insulins

**DOI:** 10.1186/s12884-016-1177-4

**Published:** 2017-01-18

**Authors:** Elisabeth R. Mathiesen, Henning Andersen, Sofia I. I. Kring, Peter Damm

**Affiliations:** 1 Department of Endocrinology, Center for Pregnant Women with Diabetes, Rigshospitalet, Institute of Clinical Medicine, Faculty of Health and Medical Sciences, University of Copenhagen, DK-2100 Copenhagen, Denmark; 2 Novo Nordisk A/S, Bagsværd, Denmark; 3 Epidemiology Department, Global Development, Novo Nordisk A/S, Bagsværd, Denmark; 4 Department of Obstetrics, Center for Pregnant Women with Diabetes, Rigshospitalet, Institute of Clinical Medicine, Faculty of Health and Medical Sciences, University of Copenhagen, Copenhagen, Denmark

**Keywords:** Diabetes, Pregnancy, Insulin detemir, Cohort study

## Abstract

**Background:**

There are a lack of data regarding the effect of basal insulin analogues on rates of events like congenital malformation and perinatal mortality in diabetic pregnancy.

**Methods:**

The present study is a prospective, non-interventional, multicentre cohort study conducted in seven countries, designed to assess the safety of insulin detemir during pregnancy, and to monitor the health status of resulting infants (exposed in utero) up to 1 year of age. The study population includes women with type 1 or type 2 diabetes, who are pregnant and being treated with insulin. Data will be collected in the context of routine practice. The primary endpoint is the proportion of pregnancies in women treated with insulin detemir, compared with other basal insulin regimens, which do not result in any of the following events: major congenital malformations, perinatal death or neonatal death. A sample size of 3075 pregnancies was calculated to provide an 80% power to detect a difference of 3.5% between groups in the primary endpoint at a 5% level.

**Discussion:**

The study will also examine other important maternal endpoints (e.g., incidences of severe hypoglycaemia and pre-eclampsia) and perinatal outcomes such as overweight neonates, as well as infant outcomes at 1 year of age. It has a fixed recruitment period from 2013 to 2018, enrolling all eligible patients, and is expected to inform future prescribing with basal insulins in diabetic pregnancy.

**Trial registration:**

ClinicalTrials.gov: NCT01892319 (date registered: 27.06.2013).

**Electronic supplementary material:**

The online version of this article (doi:10.1186/s12884-016-1177-4) contains supplementary material, which is available to authorized users.

## Background

Pregnancy in women with diabetes is associated with an increased risk of complications for the mother and the fetus/newborn. The most severe of these include congenital malformations, as well as perinatal and neonatal mortality.

Three recent nationwide population-based studies conducted in Scandinavia have shed light on the risk of such events in pregnancies among women with type 1 diabetes (T1D) [1–3]. Odds ratios (ORs) of stillbirth (ORs: 3.3 in one study and 3.6 in another), perinatal mortality (ORs: 3.3 in one study and 2.9 in another) and infant death in the first year of life (OR: 1.9) were all shown to be significantly higher among T1D than non-T1D pregnancies [1, 3]. Odds ratios of congenital abnormalities were also significantly elevated (ORs: 2.5 in one study and 2.1 in another) [1, 2].

Furthermore, in a prospective Danish multicentre study of >1000 T1D pregnancies, the perinatal annual mortality rate was 3.1% in women with T1D compared with 0.75% in the background population (OR: 4.1), and the annual rate of congenital malformation was 5.0%, compared with 2.8% in the background population (OR: 1.7) [4]. In this study, serious adverse outcomes (perinatal death and/or congenital malformations) were associated with higher glycated haemoglobin (HbA_1c_) levels before and during pregnancy [4]. Furthermore, even small elevations in HbA_1c_ in pregnant women with diabetes have been associated with significantly increased rates of congenital malformations [5]. Hence, normoglycaemia should be the target in pregnant women with diabetes and in those planning pregnancy. To achieve this, insulin will be required in all T1D patients, and in many type 2 diabetes (T2D) patients. A literature review revealed that available data on the use of basal insulins (including long-acting insulin analogues and neutral protamine Hagedorn [NPH] insulin) in pregnant women with diabetes come largely from retrospective studies [6]. These data suggest that basal insulins can be effective and safe in women with T1D during pregnancy. In a recent retrospective analysis of 113 T1D pregnancies in which women used insulin detemir (IDet) or insulin glargine (IGlar), glycaemic control and pregnancy outcomes were comparable between the two groups with regard to congenital malformations, as well as perinatal and neonatal mortality. There were no perinatal deaths and two offspring (2%, one in each group) were born with a major congenital malformation [7].

However, there are relatively few prospective data on insulin use in pregnancy. Only one randomised controlled trial (RCT) has been performed investigating the use of a basal insulin analogue: a trial comparing IDet with NPH insulin, both in combination with insulin aspart, in pregnant women with T1D [8]. In this study, IDet resulted in significantly lower fasting plasma glucose (FPG) in pregnancy compared with NPH insulin, with similar HbA_1c_ levels and rates of hypoglycaemia [8]. This trial also suggested that both insulins were well tolerated with regard to perinatal outcomes [9].

While these data are valuable, RCTs are not the best method for evaluating the occurrence of rare events like congenital malformations and perinatal/neonatal mortality. Population reports have reported outcome incidences, but not in relation to specific treatments [10–13]. Instead, large observational studies are required to assess safety and to attempt to detect this type of event, hence, such a study is warranted.

In addition to this clinical need, the European Medicines Agency (EMA) has requested that a post-authorisation safety study (PASS) be performed to monitor the long-term safety of IDet in pregnant women, covering both gestation and lactation.

The present study was therefore initiated to evaluate the safety of IDet in pregnant women, with a focus on the occurrence of malformations and perinatal/neonatal death. It will also examine other important maternal endpoints (e.g., incidences of hypoglycaemia and pre-eclampsia) and perinatal outcomes (e.g., incidences of overweight neonates/large for gestational age [LGA]), as well as infant outcomes at 1 year of age.

## Methods/design

This study is an international, prospective, non-interventional, multicentre cohort study to monitor and assess the safety of IDet in comparison with other types of insulin treatment during pregnancy, and to monitor the health status of the resulting infants (who were exposed in utero) at 1 month and 1 year of age. The large-scale data collection undertaken in this study will allow comparison and analysis of the occurrence of events between the various insulin treatment regimens. The same parameters will also be monitored for other injectable antidiabetic treatment regimens used during pregnancy.

The study is non-interventional. The assignment of patients to a particular therapeutic strategy is not decided in advance by a trial protocol but instead falls within current practice. Data will be collected in the context of routine practice, with very few exclusion criteria, and hence the results are expected to be more broadly applicable to the real-world population than those of typical RCTs.

The primary objective is to estimate the proportion of pregnancies in women treated with IDet, compared with other basal insulin regimens, which do not result in any of the following events: major congenital malformations, perinatal death or neonatal death (assessed at up to 4 weeks after delivery). The definitions of these and other key terms, within the context of this trial, are provided in Table 1.

**Table 1:** Definitions of key terms

Term	Definition
Congenital malformation	A morphological defect of an organ, part of an organ or larger region of the body resulting from an intrinsically abnormal developmental process
Foetal macrosomia	Birth weight above 4 kg
Large for gestational age	Live born infant with birth weight >90th percentile for gestational age and sex according to local reference
Major malformation	A life-threatening structural anomaly or an abnormality likely to cause significant impairment of health or functional capacity and which needs medical or surgical treatment; examples are abnormalities likely to lead to serious handicap or likely to lead to major cosmetic defects (e.g., cleft lip) and which may require major surgery to repair (e.g., atrial septal defect or ventricular septal defect)
Major hypoglycaemia	A hypoglycaemic episode in which the patient is not able to treat themselves and in which oral carbohydrates, glucagon or intravenous glucose has to be administered to the patient by another person because of severe central nervous system dysfunction
Neonatal death	Death of an infant between 7 and 28 completed days after delivery
Perinatal death	Death of a foetus/infant at ≥22 completed gestational weeks and <1 completed week after delivery
Preterm delivery	Delivery before 37 completed gestational weeks
Pre-eclampsia	A condition in pregnancy characterised by new onset of abrupt hypertension (≥140/90 mmHg documented on two occasions, ≥6 h and ≤7 days apart) and proteinuria/albuminuria
Spontaneous abortion	A naturally occurring termination of a pregnancy before 22 completed gestation weeks

The study has a fixed recruitment period of 5 years (September 2013 to September 2018), and will include gestation and follow-up of infants at 1 month and 1 year of age. The planned enrolment is 3055 patients.

The study population will include women with T1D or T2D (as assessed from maternal medical history) who are pregnant and being treated with insulin or other injectable antidiabetic treatment regimens, and who have not changed their basal insulin or other injectable antidiabetic treatment for 4 weeks prior to and following conception. Inclusion and exclusion criteria are listed in Table 2.

**Table 2:** Inclusion and exclusion criteria

Inclusion criteria
• Woman with a positive pregnancy test
• Type 1 diabetes or type 2 diabetes, diagnosed prior to conception
• Treated with unchanged basal insulin or other injectable antidiabetic treatment (for those not treated with basal insulin) for 4 weeks prior to and following conception
• Informed consent obtained before any data collection
Exclusion criteria
• Women who have been pregnant for >12 weeks at baseline visit confirmed by ultrasound

### Study endpoints

The primary endpoint is the proportion of pregnancies in women treated with IDet, compared with other basal insulin regimens, which do not result in any of the following events: major congenital malformations, perinatal death or neonatal death (assessed at up to 4 weeks after delivery).

Key secondary endpoints relate to outcomes with IDet versus other basal insulin regimens for the mother, the fetus and the infant at 1 year of age. Maternal endpoints include the incidence of major hypoglycaemia during pregnancy, the proportion of pregnancies complicated by pre-eclampsia and metabolic control (measured as HbA_1c_). Perinatal outcome endpoints include the proportion resulting in fetal macrosomia, LGA, preterm delivery or spontaneous abortion. Infant endpoints assessed at 1 year of age include height, weight, the proportion with diabetes, and the proportion with changes (progression/regression) of major congenital malformations.

Safety data will also be collected in the pregnant women participating in the study, as well as in their offspring up to 1 year of age.

For analyses of the primary and secondary endpoints, only women treated with basal insulin will be included. However, information on all insulins and other injectable antidiabetic treatment regimens will be collected to facilitate comparison and analysis of outcomes in IDet-treated patients versus, for example, patients treated with an insulin pump.

Any change in basal insulin after the baseline visit will lead to exclusion from the statistical analyses of the primary and secondary endpoints, but not from the study. However, changes in dosage or dose interval, or add-on/removal of bolus insulin, oral antidiabetic drugs and/or glucagon-like peptide-1 (GLP-1) agonists, will not lead to exclusion from the analyses.

### Treatment protocol and follow-up procedures

The frequency of visits will be determined by the individual study sites, and the data will be gathered as part of standard local routine. Only data collection at 1 month and at 1 year follow-up will be performed separately for the purpose of the study.

**Fig. 1:**
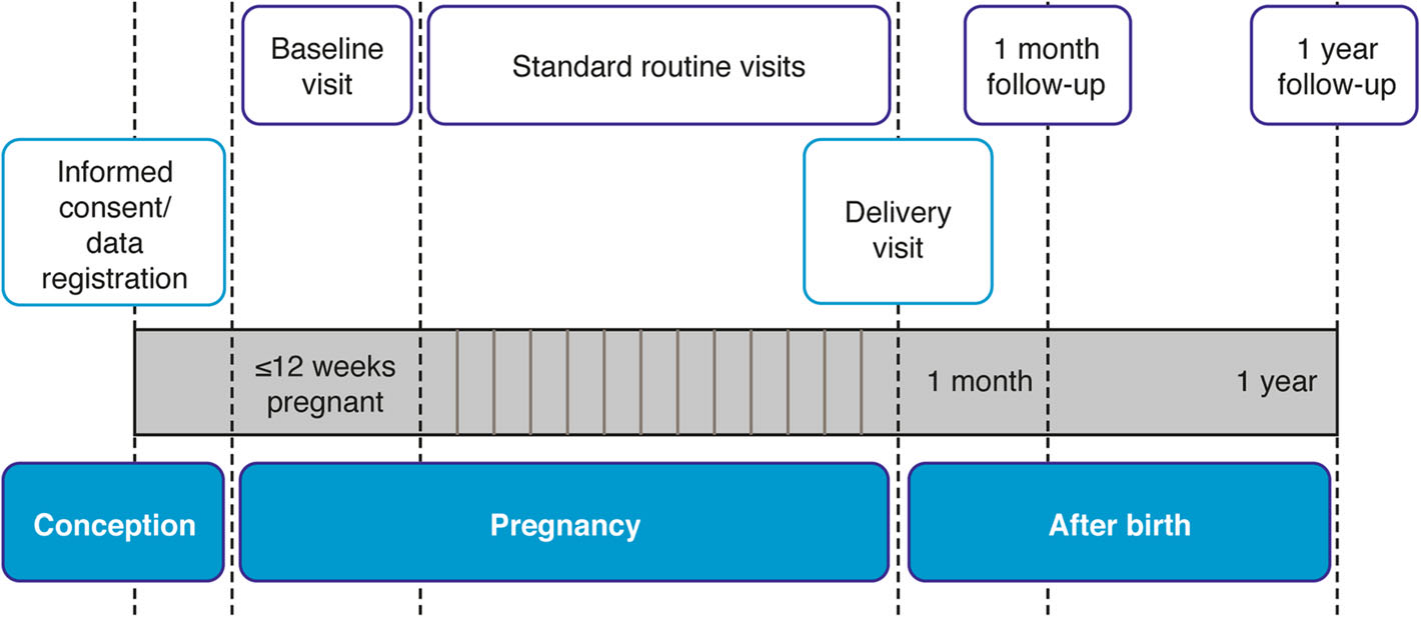
Study roadmap for each subject

Data will be collected at various time points, including baseline (occurring as soon as the woman has discovered her pregnancy through a positive pregnancy test), standard routine visits throughout pregnancy, delivery (relating both to the mother and the neonate), 1-month follow-up and end of study (1-year follow-up) (Fig. 1). Postpartum follow-up at 1 month and 1 year will be performed using questionnaires (see Additional files 1 and 2) and telephone interviews. The 1-month follow-up will collect information on height, weight, neonatal death (yes or no), congenital malformations not detected at delivery, duration of exclusive/partial lactation and any adverse events (AEs) relating to the neonate. The 1-year follow-up will collect information on the duration of exclusive and any lactation, height, weight, diabetes in the infant (yes or no), changes in major congenital malformations and any AEs relating to the infant.

### Statistical considerations

The analysis set will consist of all pregnancies among patients who are treated with basal insulin and have not changed basal insulin product for 4 weeks prior to conception and during pregnancy. Continuous variables will be summarised with descriptive statistics, and categorical variables will be displayed in frequency tables.

The primary endpoint will be analysed using a logistic regression model that will include treatment group, study site, race, hypertension, major hypoglycaemic events during pregnancy, folic acid intake and use of GLP-1 agonists during pregnancy as factors. HbA_1c_ at the start of pregnancy, the end of the first trimester and the end of the second trimester, age and body mass index at baseline, duration of diabetes, blood pressure and previous perinatal complications will be included as covariates. The primary endpoint will be computed for the IDet group and for the ‘other basal insulin’ group and the absolute difference between these proportions calculated, along with the 95% CI and the *p*-value using Fisher’s exact test.

For infant endpoints at 1 year, the proportions will also be analysed using logistic regression. Factors and covariates will be as per the primary endpoint. Analyses regarding height and weight will also be adjusted for exclusive and/or partial lactation period. Similar analyses will be performed for all other endpoints that are proportions.

Means of the continuous endpoints will be compared between the two groups using an analysis of covariance model.

The incidence of major hypoglycaemia during pregnancy will be computed for both treatment groups.

Observations of possible clinical importance (such as the primary endpoint split out according to diabetes type, insulin analogue or use of an insulin pump) will be presented using descriptive statistics.

All statistical analyses will be performed using appropriate statistical software, and tests will be performed as two-sided tests with a significance level of 0.05.

### Determination of sample size

The sample size calculation was derived from the primary endpoint, based on patients with none of the three individual outcomes: major congenital malformations, perinatal death or neonatal death. The probability of this combination of endpoints is not known within the literature. However, based on data from a UK population study [10], which are in line with results from other European countries [1, 2, 4, 11–13], the proportion of pregnancies with any of the above outcomes is estimated at 8.2%: major congenital malformations, 4.5%; perinatal death, 3%; neonatal death, 0.9% (8.2% is computed under the assumption that the individual outcomes are independent). The proportion of pregnancies *without* any of the above outcomes is therefore estimated at 91.8%.

The goal is to be able to detect a difference of 3.5% between the proportions without the combined endpoint in the IDet group compared with the other basal insulin regimens group. Assuming a maximum 1:2 split between the two groups (IDet group: other basal insulin regimens group), a sample size of 1833 pregnancies is needed to have 80% power of achieving significance at a 5% level.

To achieve a total of 1833 pregnancies for the assessment of the primary endpoint, the planned number of patients to be included during the 5 years of the recruitment period is 3075. This number is based on the expectation that ~20% of pregnancies will result in a spontaneous abortion, ~10% will be treated with other antidiabetic injectables or will change basal insulin treatment and ~10% will be lost to follow-up, leaving 60% of pregnancies (i.e., 1833) assessable for the primary endpoint.

### Study organisation

**Fig. 2:**
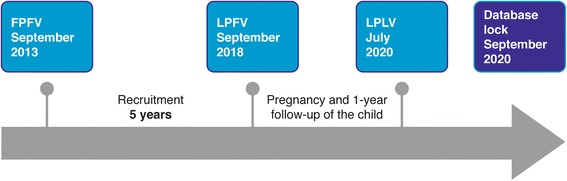
Study timeline. FPFV, first patient first visit; LPFV, last patient first visit; LPLV, last patient last visit

The study is being conducted across approximately 53 study sites in 14 countries. The anticipated numbers of patients per country are as follows: Croatia, 355; Denmark, 620; Finland, 100; France, 150; Germany, 135; Israel, 350; Italy, 130; Netherlands, 100; Poland, 200; Romania, 60; Spain, 375; and UK, 300. Additional countries and study sites might be included during the study period. Sites have relatively similar standard routine procedures with regard to the treatment of women with diabetes during pregnancy and have a relatively high prescription rate of IDet. The projected study timeline is given in Fig. 2.

The study is sponsored by a research grant from Novo Nordisk A/S. A Novo Nordisk A/S internal safety committee will perform ongoing safety surveillance. The study will be conducted in accordance with Good Pharmacoepidemiology Practice and the Declaration of Helsinki, and has been approved separately in each of the participating countries (national health authorities/local institutional review boards/independent ethics committee). Written informed consent is required from all study participants. The trial registration number is NCT01892319 (www.clinicaltrials.gov).

## Discussion

Outside of pregnancy, long-acting insulin analogues improve glycaemic control, and are associated with lower rates of hypoglycaemia than human insulins [14, 15]. Among the insulin analogues, the European Union labels for both IDet and IGlar allow for consideration of use in pregnancy [16, 17]. In the case of IGlar, this is based on postmarketing data; for IDet, an RCT has been performed, demonstrating significantly lower FPG and non-inferior HbA_1c_ in late pregnancy and similar rates of hypoglycaemia compared with NPH insulin [7].

However, none of these studies were sufficiently large to accurately evaluate the incidence of events such as congenital malformations and perinatal/neonatal mortality in patients receiving insulin during pregnancy. Hence, this long-term, prospective, epidemiological study was initiated-the first of its kind in pregnant women with diabetes.

Although the primary endpoint is based around the incidence of malformation and mortality, the study will also examine a number of other important endpoints. For example, data on incidences of pre-eclampsia, fetal macrosomia and LGA will be collected. Each of these has been shown to occur more frequently in T1D than control pregnancies. Indeed, in a Swedish population study, odds ratios were 4.5 for pre-eclampsia and 11.4 for LGA [1].

Data on the incidence of maternal hypoglycaemia will also be examined in the present study, and compared between different insulin treatments. Severe hypoglycaemic episodes are common in diabetic pregnancy, particularly in the first trimester, and are associated with a variety of factors, including impaired hypoglycaemia awareness, long duration of diabetes, fluctuating plasma glucose values and excessive use of supplementary insulin injections between meals [18].

Furthermore, the study will also examine the health of the infant at 1 year of age, including the proportion with progression or regression of major congenital malformations, as well as early growth (height and weight) and breastfeeding history. Data on such outcomes are currently lacking, and so this trial is expected to fill an important gap in understanding.

The very liberal inclusion and exclusion criteria of this study mean that the population will be largely unselected, as compared with the highly selected populations typically eligible for RCTs. The results will therefore be more broadly applicable to the real-world population of pregnant women with diabetes. Furthermore, as the study collects data based on standard routine care, participation will be substantially less demanding on the women recruited, as compared with RCTs.

In summary, this is an international, prospective, non-interventional, multicentre, cohort study designed to assess the safety of IDet in comparison with other types of insulin treatment during pregnancy, and to monitor the health status of the resulting infants - who were exposed in utero - up to 1 year of age. The planned enrolment of 3075 patients is sufficiently large to allow comparison of the frequency of rare events, such as congenital malformations and perinatal/neonatal death, between the various insulin treatment regimens. The study will therefore provide valuable information on these events, as well as other relevant maternal, pregnancy and infant-related endpoints, to inform future prescribing with basal insulins in diabetic pregnancy.

## Additional files

Additional file 1: 1-month questionnaire. (PDF 131 kb)
Additional file 2: 12-month questionnaire. (PDF 136 kb)
