# Supplementary material for: Design and rationale of a large, international, prospective cohort study to evaluate the occurrence of malformations and perinatal/neonatal death using insulin detemir in pregnant women with diabetes in comparison with other long-acting insulins
Source: BMC Pregnancy Childbirth. 2017 Jan 18;17:38. doi: 10.1186/s12884-016-1177-4 (PMC5241954; doi:10.1186/s12884-016-1177-4)
Supplement: Additional file 1: — 1-month questionnaire. (PDF 131 kb) [file 12884_2016_1177_MOESM1_ESM.pdf]

Pregnancy Registry Study

Study ID: NN304-4016

Dear Madam,

This questionnaire contains questions about the health of your child at one month of age. We kindly ask you to complete the questionnaire before your scheduled contact with your physician so that you are prepared for the questions your physician will ask you. Your answers will help us monitor and assess the health status of your child.

Thank you for taking the time to complete this questionnaire. Your answers are highly appreciated!

QUESTIONNAIRE
1 month after you gave birth

Study doctor name: ____

Study coordinator name: ____

Clinic telephone no.: ✆ ____

Patient no.: ___ ___ ___ ___ ___ ___

Infant no (in case of more than one child).: ___ ___ ___ ___ ___ ___ ___ ___

Please record the following, so that you are ready for the contact:

- Height and weight of your child
- Duration of breast feeding
- Any medical problems with your child during the first month

Date of contact (1-month follow-up):

Date: ____________________ Time: __________

novo nordisk®

Body measurements of your child:		
Your child's length at approximately one month of age:	d d m m m y y y y	. cm/in
Your child's weight at approximately one month of age:	d d m m m y y y y	. kg/lb

Duration of your breast feeding period:	Start date	Stop date, if applicable
Duration of **exclusive** breast feeding (your child is only fed breast milk)	d d m m m y y y y	d d m m m y y y y
Duration of **partial** breast feeding (your child is still fed breast milk, but also receive other nourishment (formula and/or solid food):	d d m m m y y y y	d d m m m y y y y

novo nordisk®

Medical problems for your child

Please record the following:

- Any medical problems your child has had since birth (examples: inflammation of the eye, sore throat, flu)
- Any medication your child has been given in connection with any of these medical problems
- Your own medication for this period in case you were breast feeding at the time of the event.

Examples:

Medical problems with your child			Medication for your child			Your own medication (only if you are breast feeding at the time of the event)		
**Medical problem**	**Start date**	**Stop date**	**Name of medication**	**Start date**	**Stop date**	**Name of medication**	**Start date**	**Stop date**
Inflamma-tion of eye	*18-Jun-2013*	*21-Jun-2013*	*Chloram-phenicol*	*18-Jun-2013*	*25-Jun-2013*	*Paraceta-mol*	*19-Jun 2013*	*19 Jun 2013*
Sore throat	*20-Jul-2013*	*25-Jul-2013*	*Penicillin*	*20-Jul-2013*	*30-Jul-2013*	*N/A*	*N/A*	*N/A*
Flu	*22-Aug 2013*	*27 Aug-2013*	*N/A*	*N/A*	*N/A*	*Simvastatin*	*30 Jul 2012*	*ongoing*

novo nordisk®

Medical problems with your child			Medication for your child			Your own medication (only if you are breast feeding at the time of the event)		
Medical problem	Start date	Stop date	Name of medication	Start date	Stop date	Name of medication	Start date	Stop date

novo nordisk®

Medical problems with your child			Medication for your child			Your own medication (only if you are breast feeding at the time of the event)		
Medical problem	Start date	Stop date	Name of medication	Start date	Stop date	Name of medication	Start date	Stop date
